# DDAH1 Protects against Cardiotoxin-Induced Muscle Injury and Regeneration

**DOI:** 10.3390/antiox12091754

**Published:** 2023-09-13

**Authors:** Fei Feng, Bingqing Cui, Li Fang, Ting Lan, Kai Luo, Xin Xu, Zhongbing Lu

**Affiliations:** 1 School of Exercise and Health, Shanghai University of Sport, Shanghai 200438, China; fengfei@sus.edu.cn; 2 College of Life Science, University of Chinese Academy of Sciences, Beijing 100049, China; cuibingqing18@mails.ucas.ac.cn (B.C.); luokai20@mails.ucas.ac.cn (K.L.); 3 Department of Endocrinology, Dongtai Renmin Hospital, Dongtai 224233, China; jiangsudtcdj@163.com

**Keywords:** DDAH1, cardiotoxin, muscle regeneration, oxidative stress

## Abstract

Nitric oxide (NO) is an important biological signaling molecule affecting muscle regeneration. The activity of NO synthase (NOS) is regulated by dimethylarginine dimethylaminohydrolase 1 (DDAH1) through degradation of the endogenous NOS inhibitor asymmetric dimethylarginine (ADMA). To investigate the role of DDAH1 in muscle injury and regeneration, muscle-specific *Ddah1*-knockout mice (*Ddah1*^MKO^) and their littermates (*Ddah1*^f/f^) were used to examine the progress of cardiotoxin (CTX)-induced muscle injury and subsequent muscle regeneration. After CTX injection, *Ddah1*^MKO^ mice developed more severe muscle injury than *Ddah1*^f/f^ mice. Muscle regeneration was also delayed in *Ddah1*^MKO^ mice on Day 5 after CTX injection. These phenomena were associated with higher serum ADMA and LDH levels as well as a great induction of inflammatory response, oxidative stress and cell apoptosis in the gastrocnemius (GA) muscle of *Ddah1*^MKO^ mice. In the GA muscle of CTX-treated mice, *Ddah1* deficiency decreased the protein expression of M-cadherin, myogenin, Bcl-2, peroxiredoxin 3 (PRDX3) and PRDX5, and increased the protein expression of MyoD, TNFα, Il-6, iNOS and Bax. In summary, our data suggest that DDAH1 exerts a protective role in muscle injury and regeneration.

## 1. Introduction

Skeletal muscle is the most dynamic and important organ of the body and accounts for ~40% of the body’s total weight [1]. Although exercise is good for human health, several types of muscle injury occur in people when participating in high-demand sports, including laceration, contusion and strain. It has been estimated that muscle injury accounts for 10 to 55% of all acute sports injuries [2]. Muscle has a strong ability to regenerate after injury, which is particularly important for the recovery of its function. Muscle satellite cells are skeletal muscle mononuclear progenitor cells located between the sarcolemma and the basal layer [3,4]. The process of muscle regeneration is tightly regulated by the activation of satellite cells, proliferation, differentiation and fusion into myoblasts. Studies have shown that the inflammatory response [5,6], oxidative stress [7] and apoptosis are important regulators of muscle regeneration after injury.

Cardiotoxin (CTX) is originally derived from *Naja pallida* and has been widely used as a myotoxic agent. It has been well documented that intramuscular injections of CTX induce a transient and reproducible acute muscle injury by affecting membrane calcium binding sites and lowering calcium-modulated calcium ion release from the sarcoplasmic reticulum in muscle cells which then causes the destruction of myofibers [8,9,10]. In this process, the CTX-induced injury mainly occurs in muscle while the vasculature or nerves are unaffected. In addition, the harmfulness for the experimental animal is relatively low in this model [11]. Based on these advantages, CTX-induced skeletal muscle injury has been considered as a suitable model for investing the underlying mechanism for muscle regeneration following physiological injury [10,12].

Nitric oxide (NO) is an important biological signaling molecule. The release of NO in muscle might mediate satellite cell activation by changing the adhesion of satellite cells [13], thus reflecting the process of muscle regeneration. In addition, NO plays an important role in myogenesis by stimulating myoblast differentiation and promoting myoblast fusion and subsequent myotube formation [14]. Finally, NO may also indirectly promote muscle regeneration by affecting vascular regeneration after injury [15]. The endogenous nitric oxide synthase (NOS) inhibitor, asymmetric dimethylarginine (ADMA) competes with L-arginine for binding with NOS, resulting in a decrease in NO production [16]. Under pathological conditions, ADMA can even act as a substrate for uncoupled NOS to generate a superoxide [17]. Therefore, ADMA accumulation is closely associated with endothelial dysfunction and ADMA has been regarded as a strong and independent risk factor for cardiovascular disease [18]. In vivo, ADMA is mainly degraded by dimethylarginine dimethylaminohydrolase-1 (DDAH1) [19]. Hereditary DDAH1 gene deletion or pharmacological inhibition of DDAH1 function could lead to ADMA accumulation and decreased NO signal transduction [19,20]. Therefore, DDAH1 may regulate the muscle regeneration process through NO generation.

At present, the specific effects of DDAH1 on muscle injury and regeneration have not been investigated. Hence, muscle-specific-*Ddah1* deficient mice and their littermates were used to investigate the effects of muscle *Ddah1* deletion on CTX-induced muscle injury and subsequent muscle regeneration.

## 2. Materials and Methods

### 2.1. Reagents and Antibodies

CTX and dihydroethidium (DHE) were obtained from MedChemExpress LLC (HY-P1902A, Princeton, NJ, USA) and Sigma (D7008, St. Louis, MO, USA), respectively. Wheat germ agglutinin (WGA) was purchased from Biotium Inc. (29022, Fremont, CA, USA). The ADMA ELISA kit and TUNEL staining kit were purchased from Bio-Techne Co., Ltd. (NBP2–66728, Minneapolis, MN, USA) and Beyotime Institute of Biotechnology (C1090, Shanghai, China), respectively. Lactate dehydrogenase (LDH) assay kits were obtained from Nanjing Jiancheng Bioengineering Institute (A020-2-2, Nanjing, China). Antibodies against DDAH1, peroxiredoxin 3 (PRDX3), PRDX5 and β-tubulin were purchased from Signalway Antibody LLC (37368, 38567, 38828, 48659, Greenbelt, MD, USA). Antibodies against Bcl-2, Bax, DDAH2, Interleukin-6 (IL-6), M-cadherin and TNFα were purchased from Abcam (ab194583, ab182733, ab184166, ab259341, ab129078 and ab183218, Cambridge, UK). Antibodies against F4/80 and iNOS were purchased from Wuhan Sanying Biology Technology Company (28463-1-AP and 22226-1-AP, Wuhan, China). Antibodies against MyoD and myogenin were obtained from Santa Cruz Biotechnology, Inc (sc-32758 and sc-12732, Dallas, TX, UA).

### 2.2. Animal and Experimental Design

The LoxP/Cre approach was used to obtain the muscle-specific deletion of *Ddah1* mice. The *Ddah1*^f/f^ strain was generated by introducing two loxP sites into the 3rd and 4th intron of *Ddah1* gene [21]. In the muscle creatine kinase (MCK)-Cre strain, the expression of Cre recombinase gene was driven by the *Mck* gene promoter and Cre activity could only been observed in skeletal and cardiac muscle [22]. *Ddah1*^f/f^ mice and MCK-Cre mice were kindly provided by Professor Yingjie Chen from the University of Minnesota and Professor Yan Zhang from Peking University, respectively.

To induce muscle injury, mice at the age of 8–10 weeks were administered CTX (100 μL, 10 μM) via gastrocnemius muscle injection. Control mice were injected with an equal volume of saline. Mice were anesthetized with CO_2_ and then euthanized via spinal cord dislocation on Days 3 and 5 after CTX injection. Mice were kept in individually ventilated cages with corn cob bedding and had free access to food and drinking water during the experimental period. The specific pathogen free (SPF) room for mouse housing was maintained at 24 °C with a 12 h/12 h light/dark cycle. All animal experiments were performed following the guidelines of the care and use of laboratory animals (Eighth edition, 2011).

### 2.3. Histopathological Analysis

Frozen muscle sections (8 μm) were stained with WGA and the cross-sectional area (CSA) was manually quantified using NIH ImageJ software (Ver 1.51-java 8, Bethesda, MD, USA). Mouse muscle sections were also stained with hematoxylin and eosin (H&E), antibodies against F4/80, DHE, and TUNEL kits to assess muscle inflammation, oxidative stress and apoptosis. During the quantification process, the group information was blind to the researchers to avoid errors from bias.

### 2.4. Quantitative Real-Time PCR Analysis

Total RNA was extracted from muscle tissues using TRIzol reagent. Then, the RNA samples were reverse-transcribed into cDNA using a PrimeScript RT reagent kit (#RR036B, TaKaRa, Otsu, Japan). The cDNA samples were then subjected to quantitative real-time polymerase chain reaction (qPCR) using the SYBR Premix Ex Taq™ II Kit (#RR820DS, TaKaRa). The primer sequences used are listed in Table 1. The relative mRNA levels of the target genes were calculated using the 2^−ΔΔCT^ method, and the results were normalized to the level of 18S ribosomal RNA.

### 2.5. Western Blotting Analysis

Muscle proteins were extracted via RIPA buffer containing 1% phenylmethanesulfonyl fluoride as well as protease and phosphatase inhibitor cocktails from Roche (04693124001, 4906837001, Basel, Switzerland). Protein concentration was determined using the enhanced BCA protein detection kit (#P0010, Beyotime, Shanghai, China). The detailed process of the Western blot was described in our previous report [23]. In brief, the protein samples were mixed with the loading buffer and boiled for 5 min. Then, the obtained samples were loaded and separated using 10% or 12% SDS–PAGE gels. When the electrophoresis was finished, the gels were transferred to polyvinylidene fluoride membranes. After that, the membranes were blocked with 5% nonfat milk dissolved in TBS-T buffer, and then incubated with the indicated primary antibodies (~1:1000) overnight. The membranes were then thoroughly washed and incubated with the corresponding horseradish peroxidase-labeled secondary antibodies (~1:10,000). To visualize the blots, the membranes were reacted with the chemiluminescent substrate and then subjected to the ChemiDoc™ XRS+ Gel Imaging System (Bio-Rad Laboratories, Inc., Hercules, CA, USA) for image acquisition.

### 2.6. Statistical Analysis

All results were expressed as the mean ± standard error of the mean (SEM). GraphPad Prism 9 Software (GraphPad Software Inc., San Diego, CA, USA) was used for data analysis. The differences in each variable between groups were compared using two-way analysis of variance (ANOVA) following a post hoc Tukey’s test. Statistical significance was defined as *p* < 0.05.

## 3. Results

### 3.1. Muscle-Specific Ddah1 Deletion Caused More Muscle Weight Loss and Severe Damage in Response to CTX

**Figure 1:**
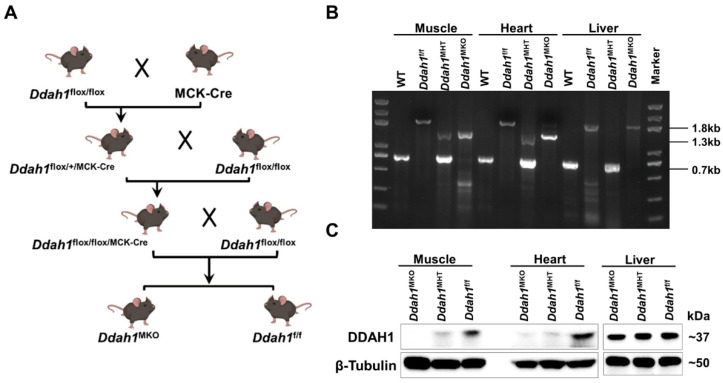
*Ddah1*^MKO^ mice were generated and identified. (**A**) The approach for *Ddah1*^MKO^ mouse generation is shown in the diagram. (**B**) The genotyping of the *Ddah1*^MKO^ mice was performed using genomic DNA extracted from muscle, heart and liver. (**C**) Muscle, heart and liver lysates were examined via Western blot. WT, wildtype; MHT, muscle heterozygous knockout.

To obtain muscle-specific *Ddah1* KO mice, *Ddah1*^f/f^ mice were crossed with MCK-Cre mice using the strategy illustrated in Figure 1A. *Ddah1*^f/f; MCK-cre/+^ (referred to hereafter as *Ddah1*^MKO^) mice were compared with littermate “flox” controls. The *Ddah1*^f/+; MCK-cre/+^ mice were heterozygote and named *Ddah1*^MHT^ mice. There are three genotyping PCR products: the 0.7 kb is for the wild type (WT), 1.3 kb is for the exon 4 deleted allele and 1.8 kb is for the exon 4 floxed allele [21]. Genomic DNA PCR showed that the exon 4 of DDAH1 was successfully deleted in the muscle and heart, but not in the liver of *Ddah1*^MKO^ or *Ddah1*^MHT^ mice (Figure 1B). Western blot analysis showed that DDAH1 expression was extremely low in the muscle and heart of *Ddah1*^MKO^ mice. However, DDAH1 expression was similar in the livers of *Ddah1*^MKO^, *Ddah1*^MHT^ and *Ddah1*^f/f^ mice (Figure 1C).

**Figure 2:**
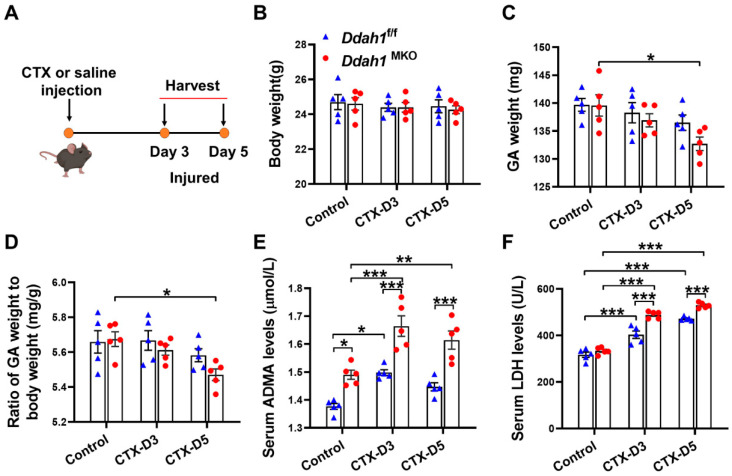
*Ddah1*^MKO^ mice exhibited more muscle weight loss and injury after cardiotoxin treatment. (**A**) Schematic diagram showing experimental design of this study. On Days 3 and 5 after cardiotoxin (CTX) injection, (**B**) body weight, (**C**) gastrocnemius (GA) muscle weight, (**D**) ratio of GA weight to body weight (GA/BW), serum asymmetric dimethylarginine (ADMA) (**E**) and lactate dehydrogenase (LDH) (**F**) levels in each group were measured. Values are expressed as the means ± SEM (*n* = 5). * indicates *p* < 0.05, ** indicates *p* < 0.01, *** indicates *p* < 0.001.

In accordance with the previous studies [24,25], 3 and 5 days after CTX injection were chosen to represent the muscle injury and muscle regeneration period, respectively (Figure 2A). CTX injection did not affect body weight, gastrocnemius (GA) weight and the ratio of GA weight to body weight (GA/BW) in *Ddah1*^f/f^ mice on Days 3 and 5 (Figure 2B,C). Although CTX also did not affect the body weight of *Ddah1*^MKO^ mice, it caused a significant reduction of GA weight and the GA/BW ratio in *Ddah1*^MKO^ mice on Day 5 after injection (Figure 2D), indicating that there was more muscle weight loss in *Ddah1*^MKO^ mice. Muscle-specific *Ddah1* deletion increased serum ADMA levels in control and CTX-treated mice. Serum ADMA levels were significantly elevated in both *Ddah1*^f/f^ and *Ddah1*^MKO^ mice on Day 3 after CTX injection, while increases in serum ADMA levels were observed only in *Ddah1*^MKO^ mice on Day 5 (Figure 2E). In addition, CTX injection increased serum LDH levels in both *Ddah1*^f/f^ and *Ddah1*^MKO^ mice on Days 3 and 5. However, *Ddah1*^MKO^ mice exhibited significantly higher serum LDH levels than *Ddah1*^f/f^ mice at each time point (Figure 2F), indicating that *Ddah1*^MKO^ mice had more muscle injury after CTX injection.

### 3.2. Muscle-Specific Ddah1 Deletion Delayed Muscle Regeneration in Response to CTX

**Figure 3:**
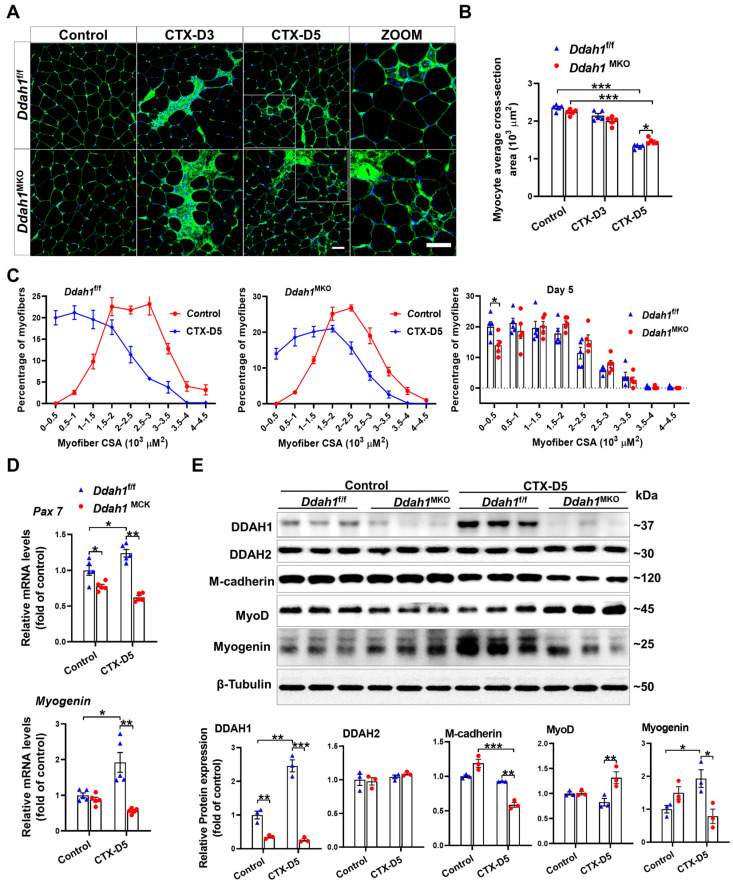
*Ddah1* affects CTX-induced muscle regeneration. (**A**) Cryosections from control and CTX–treated GA muscle were stained with wheat germ agglutinin (WGA). Scale bars = 50 μm. (**B**) The averaged myofiber cross-sectional area was measured via Image J. (**C**) GA myofiber size distribution in control and CTX–treated mice is shown. (**D**) The mRNA levels of genes involved in muscle regeneration were measured via qPCR. (**E**) GA muscle was homogenized in RIPA buffer and lysates were examined via Western blotting. In Figure (**A**–**D**), *n* = 5; in Figure (**E**), *n* = 3; values are expressed as the means ± SEM, * indicates *p* < 0.05, ** indicates *p* < 0.01, *** indicates *p* < 0.001.

To determine whether *Ddah1* affects muscle regeneration after CTX injection, myofiber area quantification was performed using WGA staining on GA muscle cryosections (Figure 3A). Five days after CTX injection, the average myofiber CSA of GA muscle in *Ddah1*^f/f^ mice was lower than that in *Ddah1*^MKO^ mice (Figure 3B). Compared with the control group, myofiber size distribution calculated from GA muscles showed a leftward shift on Day 5 after CTX injection. Small muscle fibers (0~0.5 × 10^3^ μm^2^), which have been regarded as newly formed muscle fibers, were increased in both *Ddah1*^f/f^ and *Ddah1*^MKO^ mice. However, *Ddah1*^MKO^ mice had fewer muscle fibers of a small size (Figure 3C). To further confirm the slowed muscle regeneration in *Ddah1*^MKO^ mice, the levels of genes involved in muscle regeneration were examined via qPCR. Under control conditions, *Pax 7* mRNA levels were lower in the GA muscle of *Ddah1*^MKO^ mice. CTX injection caused significant increases in *Pax 7* and *Myogenin* mRNA levels in the GA muscles of *Ddah1*^f/f^ mice, and such increases were diminished in *Ddah1*^MKO^ mice (Figure 3D). Interestingly, CTX significantly increased DDAH1 and Myogenin protein expression but did not affect DDAH2, M-cadherin and MyoD expression in the GA muscle of *Ddah1*^f/f^ mice. However, CTX had no effect on Myogenin expression but caused significant decreases in M-cadherin expression in *Ddah1*^MKO^ mice. Compared to the *Ddah1*^f/f^ mice, the protein expression of M-cadherin and Myogenin was lower, while MyoD expression was higher in the GA muscle of *Ddah1*^MKO^ mice (Figure 3E). These results suggest that muscle *Ddah1* may promote muscle regeneration after CTX injection.

### 3.3. Muscle-Specific Ddah1 Deletion Aggravated the Inflammatory Response after CTX Treatment

**Figure 4:**
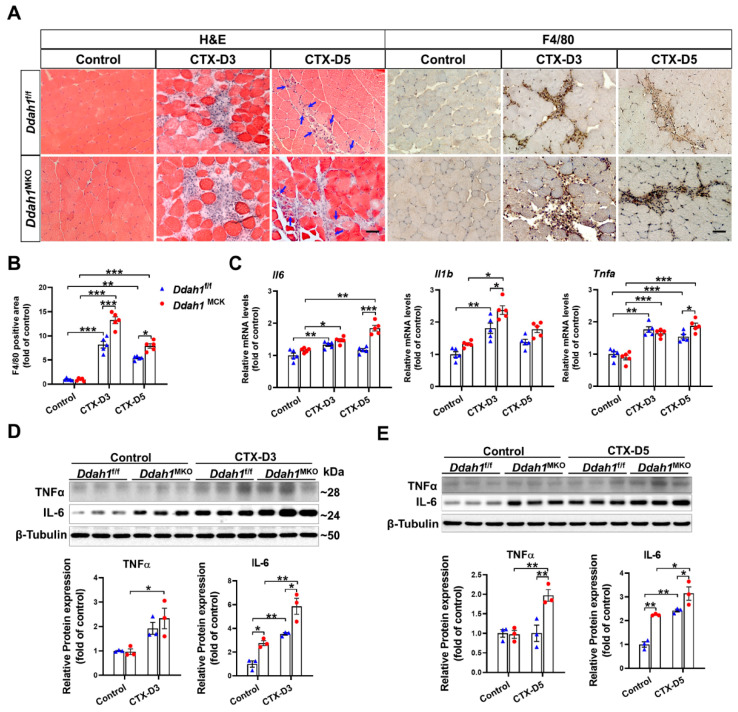
Muscle-specific *Ddah1* deletion exacerbated the CTX-induced muscle inflammatory response. (**A**) Representative GA muscle sections from control and CTX-treated *Ddah1*^f/f^ and *Ddah1*^MKO^ mice were stained with hematoxylin and eosin (H&E) and an antibody specific for macrophages (F4/80) (brown staining). Scale bar = 50 μm. (**B**) The F4/80-positive cell numbers were quantified. (**C**) The mRNA levels of inflammatory factors in each group were measured on Days 3 and 5 after CTX injection. (**D**,**E**) Muscle lysates were subjected to Western blot analysis. In Figure (**A**–**C**), *n* = 5; in Figure (**D**,**E**), *n* = 3; values are expressed as the mean ± SEM, * indicates *p* < 0.05, ** indicates *p* < 0.01, *** indicates *p* < 0.001.

Muscle regeneration after CTX injection usually starts at the late stage of inflammation, and persistent recruitment of inflammatory cells can delay the regeneration process [26]. H&E staining revealed that CTX injection caused more inflammatory cell infiltration in the GA muscles in *Ddah1*^MKO^ mice than in *Ddah1*^f/f^ mice on Day 3. On Day 5, the morphological changes and inflammatory cell infiltration in GA muscles were significantly improved in *Ddah1*^f/f^ mice but not in *Ddah1*^MKO^ mice (Figure 4A). In addition, as indicated by the blue arrow, which is a marker of muscle regeneration with the central nucleus, GA muscles from *Ddah1*^MKO^ mice exhibited less muscle regeneration on Day 5 (Figure 4A). As a mature mouse cell surface glycoprotein expressed at high levels on various macrophages, F4/80 has been regarded as a well-characterized and extensively referenced mouse macrophage-specific marker [27]. Immunohistochemical staining using an anti-F4/80 antibody showed that there was more macrophage infiltration in the GA muscle from *Ddah1*^MKO^ mice than in that from *Ddah1*^f/f^ mice on Days 3 and 5 after CTX injection (Figure 4A,B). To further determine the effect of *Ddah1* on the muscle inflammatory response, the mRNA levels of several inflammatory factors were measured. On Day 3 after CTX injection, the mRNA levels of *Il6*, *Il1b* and *Tnfa* were significantly increased in the GA muscles from both *Ddah1*^f/f^ and *Ddah1*^MKO^ mice. However, CTX caused more increases in Il1b mRNA levels in *Ddah1*^MKO^ mice than in *Ddah1*^f/f^ mice. On Day 5, increases in *Tnfa* mRNA levels were observed in both genetic mice, while the increases in Il6 mRNA levels were only significant in *Ddah1*^MKO^ mice. In addition, *Ddah1*^MKO^ mice exhibited significantly higher *Il6* and *Tnfa* mRNA levels than *Ddah1*^f/f^ mice (Figure 4C). Western blot revealed that CTX significantly increased TNFα protein expression in the GA muscle of *Ddah1*^MKO^ mice on Days 3 and 5 but not in *Ddah1*^f/f^ mice. Muscle-specific *Ddah1* deletion significantly increased IL-6 protein expression in control muscles and further exacerbated the CTX-induced upregulation of IL-6 (Figure 4D).

### 3.4. Muscle-Specific Ddah1 Deficiency Aggravated CTX-Induced Cell Apoptosis and Oxidative Stress

**Figure 5:**
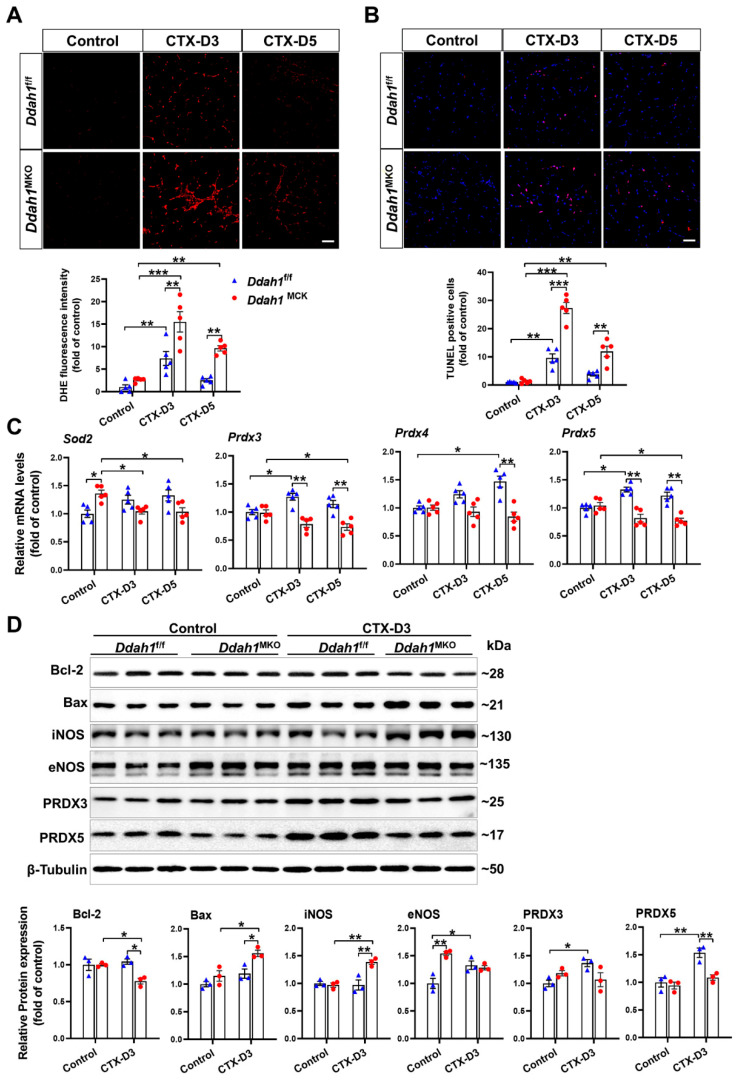
Muscle-specific *Ddah1* deletion exacerbated CTX-induced muscle oxidative stress and apoptosis. (**A**,**B**) Representative frozen muscle sections from each group were stained with DHE (**A**) and TUNEL (**B**). The relative fluorescence intensity and apoptotic cell numbers were quantified. Scale Bar = 50 μm. (**C**) The mRNA levels of related antioxidant enzymes were measured via qPCR. (**D**) The GA muscle lysates were analyzed via Western blotting. In Figure (**A**–**C**), *n* = 5; in Figure (**D**), *n* = 3; values are expressed as mean ± SEM, * indicates *p* < 0.05, ** indicates *p* < 0.01, *** indicates *p* < 0.001.

It has been reported that muscle regeneration can be delayed by excess superoxide generation [28] and that the severity of muscle injury is associated with cell apoptosis [29]. To determine the effect of DDAH1 on CTX-induced oxidative stress and apoptosis, cryosections from control and CTX-treated mice were stained with DHE and TUNEL, respectively. As shown in Figure 5A,B, CTX injection caused significant increases in superoxide levels and apoptotic cell numbers on Day 3 in the GA muscle of *Ddah1*^f/f^ and *Ddah1*^MKO^ mice. On Day 5, such increases were found only in *Ddah1*^MKO^ GA muscle. In addition, the GA muscle from *Ddah1*^MKO^ mice exhibited higher superoxide levels and more apoptotic cell numbers than that from *Ddah1*^f/f^ mice on Days 3 and 5 after CTX injection (Figure 5A,B). To investigate the underlying mechanism through which DDAH1 regulates CTX-induced oxidative stress, we measured the mRNA levels of some antioxidant enzymes via qPCR. On Day 3, CTX injection increased the mRNA levels of *Prdx3* and *Prdx5* in *Ddah1*^f/f^ mice, but decreased *Sod2* mRNA levels in *Ddah1*^MKO^ mice. The mRNA levels of *Prdx3* and *Prdx5* were significantly lower in *Ddah1*^MKO^ mice than in *Ddah1*^f/f^ mice. On Day 5, CTX caused significant decreases in *Sod2*, *Prdx3* and *Prdx5* mRNA levels in *Ddah1*^MKO^ mice but increased *Prdx4* mRNA levels in *Ddah1*^f/f^ mice. *Ddah1*^MKO^ mice exhibited significantly lower *Prdx3*, *Prdx4* and *Prdx5* mRNA levels than *Ddah1*^f/f^ mice (Figure 5C).

Since the differences in superoxide levels and apoptotic cell numbers were more significant on Day 3, the samples collected on Day 3 were analyzed using Western blotting to examine the effect of DDAH1/CTX on the expression of oxidative stress- and apoptosis- related proteins. In control mice, muscle-specific *Ddah1* deletion significantly increased eNOS protein expression. In the GA muscle of *Ddah1*^f/f^ mice, CTX injection significantly increased the protein expression of eNOS, PRDX3 and PRDX5 but did not affect the expression of iNOS, Bcl-2 and Bax. In contrast, CTX decreased Bcl-2 expression and increased iNOS and Bax expression in the GA muscle of *Ddah1*^MKO^ mice. Compared with that in *Ddah1*^f/f^ mice, the protein expression of PRDX5 and Bcl-2 was lower, whereas the expression of iNOS and Bax was higher in *Ddah1*^MKO^ mice (Figure 5D).

## 4. Discussion

There were two new findings in the present study. First, we demonstrated that muscle-specific *Ddah1* deficiency exacerbated CTX-induced muscle injury and delayed muscle regeneration. Second, the detrimental effect of muscle-specific *Ddah1* deletion on CTX-induced muscle injury was associated with increases in ADMA levels and aggravation of inflammation, oxidative stress and apoptosis.

The protective effect of DDAH1 has been observed in acute myocardial infarction [30], PM_2.5_-induced lung injury [31], acetaminophen-induced liver injury [23], high fat diet-induced hepatic steatosis [32] and destabilization of the medial meniscus surgery-induced osteoarthritis models [33]. Here, we demonstrated that CTX caused more muscle weight loss, enhanced the inflammatory response and oxidative stress, and increased serum LDH levels and apoptotic cell numbers in *Ddah1*^MKO^ mice. Moreover, there were fewer newly formed myofibers and fused nuclei in the GA muscle of *Ddah1*^MKO^ mice on Day 5 after CTX injection. These results suggested that DDAH1 could also protect against CTX-induced muscle injury and promote muscle regeneration.

It is well-known that the muscle has an enormous capacity for regeneration after injury, which originates from the activation, proliferation and differentiation of satellite cells [34]. Previous reports have demonstrated that satellite cell activation is mediated by local NO release and that the inhibition of NOS activity delays muscle repair and regeneration [13,35]. In that regard, DDAH1 may promote muscle regeneration by degrading ADMA thereby increasing local NO production. In fact, we did show that the increased serum ADMA levels in CTX-treated *Ddah1*^MKO^ mice were associated with lower mRNA levels of Pax7 and Myogenin, which are essential transcription factors for myogenesis. The repression of Pax7 by NOS inhibitor (L-NAME) has also been found in crush injury muscle [35]. It has been reported that MyoD is a marker for activated satellite cells [36] and that M-cadherin is important for the proliferation and fusion process of satellite cells [37]. In addition, Myogenin is expressed at the beginning of myoblast differentiation and helps muscle myoblasts fuse [38]. Here, we demonstrated that muscle from CTX-treated *Ddah1*^MKO^ mice had a lower expression of M-cadherin and higher expression of MyoD on Day 5, suggesting that most of the satellite cells in the GA muscle of *Ddah1*^MKO^ mice were just activated and had not entered the rapid proliferation and subsequent fusion process on Day 5 after the CTX injection.

During the process of muscle regeneration, the initially recruited proinflammatory macrophages could enhance the proliferation but inhibit the differentiation of myogenic cells. Then, proinflammatory macrophages transform into anti-inflammatory macrophages, which promote the differentiation and fusion of myogenic progenitors [39,40,41]. We previously showed that DDAH1 inhibits inflammatory cell infiltration in PM_2.5_-exposed lungs. The anti-inflammatory mechanism of DDAH1 may be related to the inhibition of NF-κB. We previously reported that deletion of *Ddah1* in MEF cells resulted in the activation of NF-κB [42] and the loss of *Ddah1* also exacerbated NF-κB activation under stress conditions [31,32]. The present study found that *Ddah1*^MKO^ mice had more F4/80-positive macrophage infiltration and higher levels of proinflammatory factors TNFα and IL-6 than *Ddah1*^f/f^ mice on Day 5 after CTX injection, suggesting that the pro- to anti-inflammatory phenotype transition of macrophages might be attenuated by *Ddah1* deficiency, thus delaying the process of muscle regeneration.

Muscle injury and regeneration are also affected by the cellular redox state [43], and excess ROS production may cause the inhibition of myogenic differentiation [44]. Downregulation of SOD1 [28] or iron overload [45] impaired myogenesis in CTX-treated mice by increasing ROS levels. In the present study, we demonstrated that muscle-specific *Ddah1* deficiency aggravated CTX-induced superoxide generation and attenuated the induction of PRDX3 and PRDX5, suggesting that DDAH1 may promote muscle regeneration by acting as an antioxidant enzyme and/or a regulator of antioxidant enzymes. The finding that there were more TUNEL-positive cells, higher Bax expression and lower Bcl-2 expression in the GA muscle of *Ddah1*^MKO^ mice on Day 3 following CTX injection suggested that DDAH1 protects against CTX-induced muscle injury by inhibiting apoptosis. The antioxidative and anti-apoptotic effects of DDAH1 have been reported in different cell models [42], APAP-induced liver injury [23] and PM_2.5_-induced lung injury [31]. In addition, we recently showed that ADMA increases intracellular ROS levels in iNOS-overexpressing macrophages [31]. A previous study showed that the increased iNOS expression in injured muscle was mainly located in macrophages [26]. Here, the expression of iNOS was higher in the GA muscle of *Ddah1*^MKO^ mice on Day 3, which was associated with higher serum ADMA levels. Therefore, DDAH1 may also regulate the microenvironmental ROS levels by degrading circulating or local ADMA.

DDAH1 expression could be upregulated by different stresses, including acute PM_2.5_ exposure [31], tert-Butyl hydroperoxide treatment [46] and hypoxia [47]. Here, we showed that CTX significantly increased DDAH1 expression in GA muscles. Considering that DDAH1 is beneficial for muscle regeneration, the upregulation of DDAH1 might be a self-protective mechanism in response to CTX. Interestingly, exercise training is believed to promote muscle regeneration after injuries [48]. We previously observed that swimming for 8 weeks increases myocardial DDAH1 expression and promotes cardiac angiogenesis in mice [49]. Moreover, a recent study showed that exercise induced DDAH1 upregulation in bones through enhancing the binding capacity between TAZ and SMAD4 [50]. Therefore, it is possible that DDAH1 is involved in exercise-induced muscle regeneration. The effects of exercise type and time on muscle DDAH1 expression and whether DDAH1 mediates the beneficial effects of exercise should be investigated in future studies.

The present study has two limitations. First, the NO levels and NOS activity in GA muscle have not be measured. Second, the effect of DDAH1 on long-term muscle recovery has not been determined. Further studies are necessary to resolve these limitations and clarify the details of underlying mechanism of DDAH1 on muscle regeneration.

## 5. Conclusions

In summary, our data suggest that DDAH1 in muscle cells protects against CTX-induced muscle injury and promotes muscle regeneration by degrading ADMA and repressing inflammation, oxidative stress and apoptosis. These results indicate that DDAH1 might be a potential therapeutic target for acute muscle injury.

## Figures and Tables

**Table 1:** The primers used in Quantitative real-time PCR.

Genes	Gene ID	Primers	Sequence
*Pax7*	18509	Forward	5′-TCTCCAAGATTCTGTGCCGAT-3′
		Reverse	5′-CGGGGTTCTCTCTCTTATACTCC-3′
*Myogenin*	17928	Forward	5′-GAGACATCCCCCTATTTCTACCA-3′
		Reverse	5′-GCTCAGTCCGCTCATAGCC-3′
*Il6*	16193	Forward	5′-AACGATGATGCACTTGCAGA-3′
		Reverse	5′-TGGTACTCCAGAAGACCAGAGG-3′
*Il1β*	16176	Forward	5′-AGGTCAAAGGTTTGGAAGCA-3′
		Reverse	5′-TGAAGCAGCTATGGCAACTG-3′
*Tnfα*	21926	Forward	5′-CCCTCACACTCAGATCATCTTCT-3′
		Reverse	5′-CCCTCACACTCAGATCATCTTCT-3′
*Sod2*	20656	Forward	5′-CCTACGTGAACAATCTCAACG-3′
		Reverse	5′-GGCTGAAGAGCGACCTGAGTT-3′
*Prdx3*	11757	Forward	5′-CTGAGTGTCAACGACCTTCCG-3′
		Reverse	5′-ACTGGAACGCCTTTACCAAACG-3′
*Prdx4*	53381	Forward	5′-TCCTGTTGCGGACCGAATC-3′
		Reverse	5′-CCACCAGCGTAGAAGTGGC-3′
*Prdx5*	54683	Forward	5′-CCAAGTTCACCTTCTTTCCCG-3′
		Reverse	5′-GGAGATGCCATTCCCTCAGTG-3′
*18s*	19791	Forward	5′-TTCTGGCCAACGGTCTAGACAAC-3′
		Reverse	5′-CCAGTGGTCTTGGTGTGCTGA-3′

## Data Availability

The data presented in this study are available on reasonable request from the corresponding author.
